# Retrospective analysis of radiographic signs in feline pleural effusions to predict disease aetiology

**DOI:** 10.1186/s12917-022-03218-3

**Published:** 2022-03-26

**Authors:** Lily Hung, Belinda Judith Hopper, Zoe Lenard

**Affiliations:** Animalius, Diagnostic Imaging, Animalius Vet, 6 Focal Way Bayswater, Perth, WA 6053 Australia

**Keywords:** Pleural effusion, Idiopathic chylothorax, Pyothorax, Congestive heart failure, Feline pleural effusion, Radiographic parameters, Positive and negative predictive values, Mediastinal mass, Thorax radiography, Cardiomegaly

## Abstract

**Background:**

The objectives of the study were to determine the prevalence of underlying conditions causing pleural effusion in cats and to calculate the positive predictive values, negative predictive values, sensitivity and specificity of radiographic signs to predict aetiology of the pleural fluid.

**Methods:**

Data from 148 cats with pleural effusion and diagnosed with known aetiologies were retrospectively analysed. Sixty one cats had thoracic radiographs evaluated by consensus through pre-defined radiographic signs by two radiologists blinded to the diagnoses.

**Results:**

Congestive heart failure (53.4%) was the most common diagnosis, followed by neoplasia (20.3%), pyothorax (10.8%), idiopathic chylous effusion (5.4%), feline infectious peritonitis (1.4%) and "other" or cats with multiple diagnoses (total 8.8%). Cats with an enlarged cardiac silhouette had a high positive predictive value of congestive heart failure (90%). Mediastinal masses (100%)and pulmonary masses (100%) were highly predictive of neoplastic disease. Pulmonary nodules (50%) were poorly predictive of neoplastic disease. The remainder of the radiographic variables were not informative predictors of underlying disease.

**Conclusions:**

In our sample of cats, congestive heart failure was the most common cause of pleural effusion. Radiographically enlarged cardiac silhouette and presence of a mediastinal mass may be useful predictors of aetiology, however there are limitations to the use of radiography alone as a diagnostic tool.

**Supplementary Information:**

The online version contains supplementary material available at 10.1186/s12917-022-03218-3.

## Background

Pleural effusion can be a life-threatening condition caused by altered fluid dynamics leading to abnormal accumulation of fluid between the visceral and parietal pleura [1–4]. Patients with pleural space disease may show little or subtle clinical signs with gradual reduction in haemoglobin saturation [5]. By the time patients with pleural effusion present with signs of dyspnoea (open mouth breathing, agitation), there can be minimal functional reserve remaining [1]. Handling may lead to a sudden requirement in oxygen demand which can precipitate respiratory failure [1, 5]. If significant pleural effusion is identified, careful and minimal handling of the patient and immediate thoracocentesis is recommended [1, 6].

Whilst pleural effusion may be straightforward to detect, understanding the primary cause may be difficult. Common documented causes of pleural effusions in cats include feline infectious peritonitis, congestive heart failure, pyothorax, neoplasia, idiopathic chylothorax and trauma [1, 4, 6–8]. The diagnosis of these findings may include cytological assessment of pleural effusion, radiography, echocardiography and/ or computed tomography [1–3, 6].

Determining the underlying cause of effusion allows for accurate prognostication and treatment, as treatment for congestive heart failure is vastly different from treatment of neoplasia [6]. Cytological assessment of the effusion can be valuable for diagnosis but has its limitations. For example, chylous effusions are encountered in congestive heart failure, neoplasia, trauma or can be idiopathic [1, 9–11].

Radiography remains an important component of diagnostic investigation in cats with respiratory distress [1, 12–16].There are no structured investigations of the usefulness of radiographic signs in predicting disease type in cats with pleural effusions with anecdotal evidence regarding unilateral distribution of pyothorax effusions [4, 6, 8] and abnormal pleural contours in cats with chylothorax [4, 8].

The aims of this study were to determine the prevalence of underlying conditions causing pleural effusions in cats and assess sensitivity, specificity, positive and negative predictive values of radiographic signs for the various underlying aetiologies.

## Materials and methods

### Sampling strategy

Clinical records of cats with pleural effusion were recruited retrospectively from 2009 to 2020 across three referral and one primary care veterinary hospital. All veterinary hospitals were located within a 10 km radius of metropolitan Perth. A majority of the cases (*n* = 136) were obtained from a single multi-disciplinary specialist hospital (Perth Veterinary Specialists, Perth, Western Australia, Australia). Inclusion criteria were the presence of pleural effusion (confirmed on thoracic ultrasound, CT, radiography or positive thoracocentesis) with a definitive or presumptive diagnosis documented by the primary clinician after assessment of all available diagnostic data (clinical parameters, cytology, histopathology, imaging modalities, treatment response). Cats were excluded if medical records were incomplete. Data collected for the study included signalment (age, breed, sex), diagnostic imaging results (radiography, echocardiography, thoracic ultrasound, CT assessment of the thorax), fluid analysis (cytological, biochemical, fluid culture), and tissue histopathology. Due to the retrospective nature of the study, not all diagnostic tests were performed and available for every patient.

Cats were classified by underlying aetiology according to the following criteria:

1. Congestive heart failure (CHF): left atrium to aorta ratio larger than 1.6 [17, 18] and/or left atrium on right parasternal long axis view > 16.8 mm diameter [17], with the echocardiography performed by a board-certified or board-eligible radiologist, or by a resident in training with direct supervision. In some cases, measurements were taken retrospectively from the echocardiogram reports and echocardiogram images. There are no veterinary cardiology services available in Perth, Western Australia; therefore, all studies were conducted by radiologists trained in echocardiography.
2. Neoplasia: diagnosed by either obtaining cytological or histopathological evidence of neoplasia. Cytology and histopathology of neoplastic disease were assessed by board certified pathologists.
3. Pyothorax: detection of septic exudate on cytology and/or detection of intracellular bacteria on cytology or presence of a positive bacterial culture by an external Veterinary laboratory with board certified pathologists [7].
4. Idiopathic chylothorax: cytological findings on effusion (predominance of small lymphocytes and non-degenerative neutrophils with background chylomicrons) and elevated triglycerides (> 1.13 mmol/L) and/ or a cholesterol to triglyceride ratio of more than 1 [1, 19]. All cats had an echocardiograms performed which did not identify cardiac disease, and without a history of trauma or evidence of neoplasia [20]. All cytological evaluation was performed by an external Veterinary laboratory with board certified pathologists.
5. Feline infectious peritonitis: positive immunohistochemical staining of FCoV antigen in tissue macrophages or fluid cytology (direct IFA), FIP RT-PCR assays were included. Cats with highly suggestive clinical signs (one or more clinical signs of pyrexia, non-specific lethargy, weight loss and young age of less than one) with a translucent, yellow protein-rich pleural effusion (positive on Rivalta’s test) [21] was also included.
6. Other: cats not meeting the above criteria, with an alternative aetiology identified.

## Concurrent disease: two or more diseases as a potential cause of pleural effusion according to the above criteria

### Radiographic assessment

Cats with multiple diagnoses were excluded from radiographic interpretation. Studies were reviewed by all authors and radiographs with poor technique or inappropriate positioning were rejected. For inclusion into the study, patients must have had at least two orthogonal projections of the thorax of diagnostic quality. The authors understand that three orthogonal projections are recommended for thoracic interpretations; however, considering that all patients had increased respiratory effort or distress, two orthogonal views were accepted into the study. Thoracic radiographs were reviewed by two board certified veterinary radiologists (ZL, BH) that were blinded to the clinical diagnosis and read with consensus through pre-defined classification criteria. These results were used in the assessments of predictive values, sensitivity, and specificity. All radiographs were anonymised, downloaded as JPEG files and compiled into a Powerpoint slideshow (Microsoft Powerpoint; Microsoft Corporation) in a randomised order using a random number generator. A DICOM viewer was not utilised as Powerpoint was assessed to be superior for streamlining the presentation and anonymising patient details. The radiologists were able to magnify, adjust brightness and contrast of the images if desired.

**Fig. 1:**
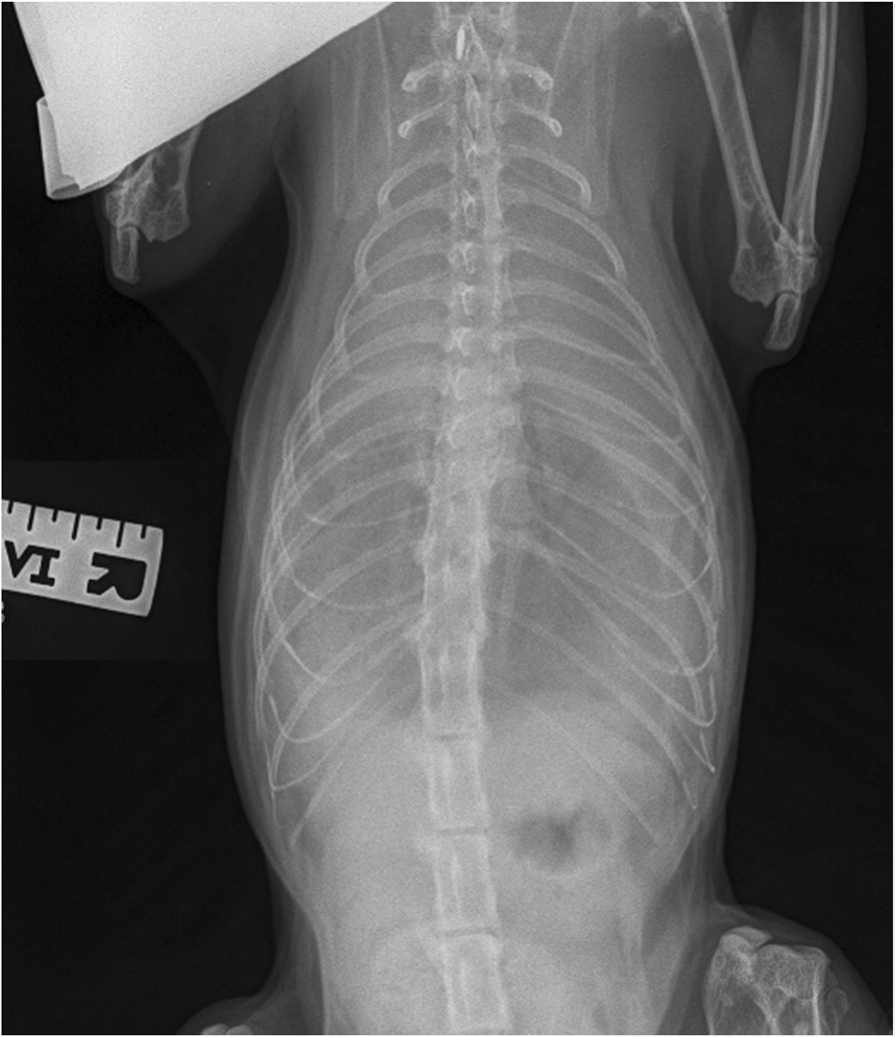
A patient with a large volume of pleural effusion with complete effacement of cardiac silhouette, indistinct pulmonary vasculature and marked difficulty assessing pleural margins and mediastinum

**Fig. 2:**
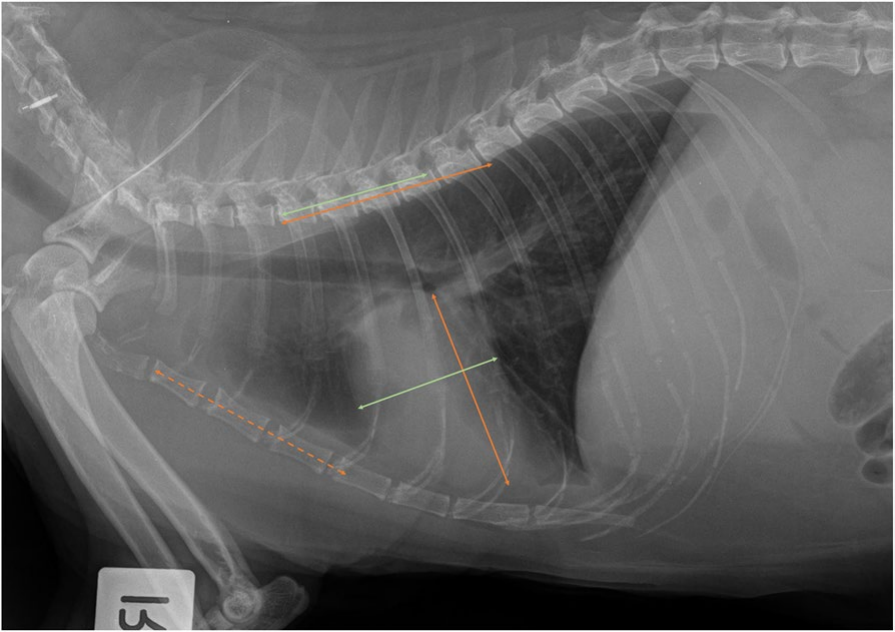
Cardiac silhouette size was measured using two methods. The first method was the vertebral heart score. The base apex length and craniocaudal length were transposed onto the vertebral column and recorded as the corresponding number of vertebrae measured from the cranial edge of T4 vertebral body. VHS more than 8 was considered enlarged and likely in heart failure. The base apex length was measured from the ventral wall of the carina to apex (orange solid line). The craniocaudal length was measured perpendicular to the base-apex length, at the widest width of the cardiac silhouette (green solid line). The second method used the base apex length and compared this against the length of sternebrae 2 to 4. If the length extended beyond three sternebrae, the cardiac silhouette was considered enlarged (dashed orange line). This patient has VHS of 9 and an elongated base-apex length suggestive of congestive heart failure

**Fig. 3:**
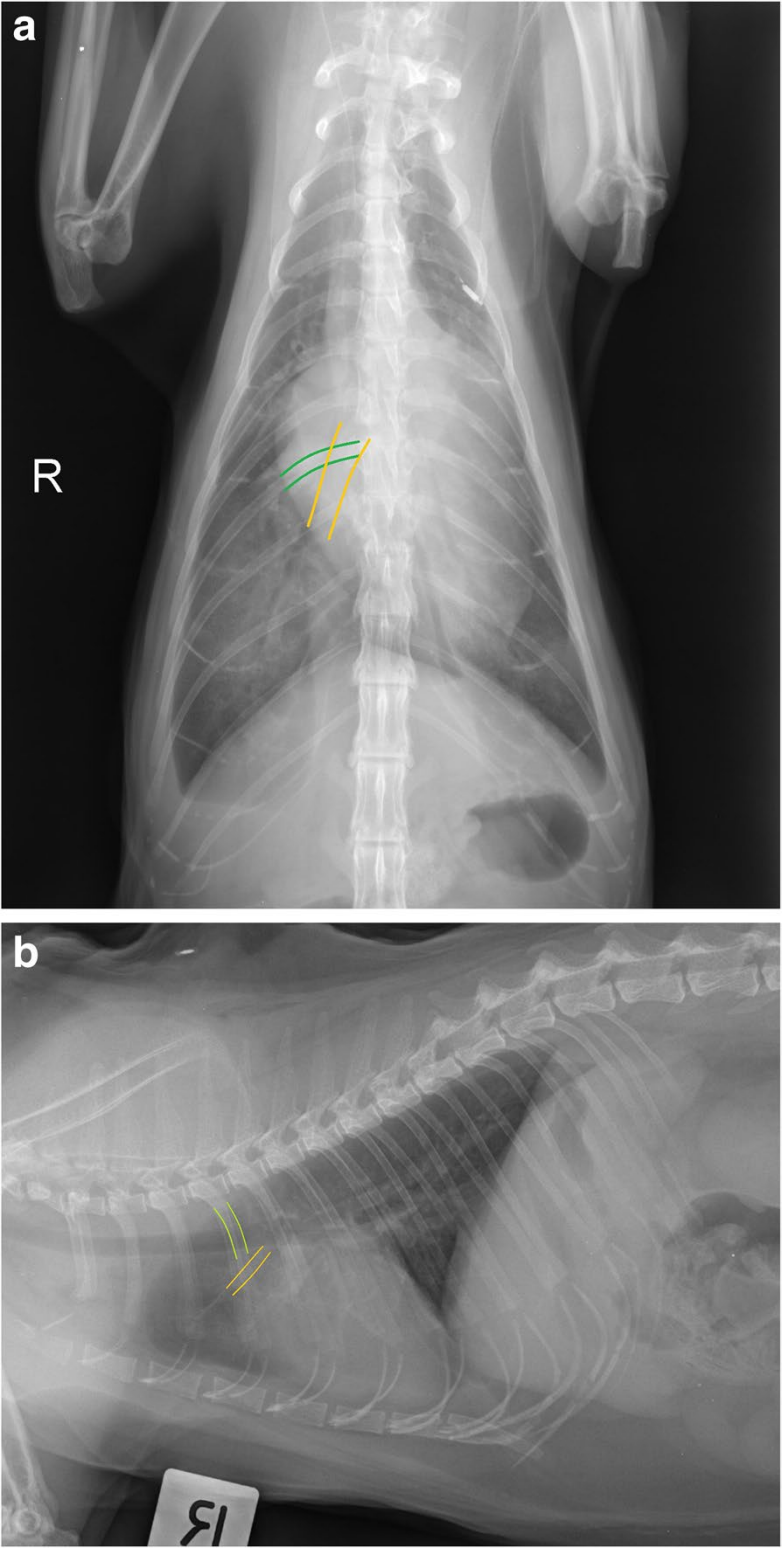
**a **VD projection. Caudal lobar vein (depicted by orange solid line) and veins at where they intersect with the 9th rib (depicted by green solid line) were considered enlarged if they were more than 1:1 ratio. This cat had bilateral enlarged caudal pulmonary veins and arteries with a scant volume of pleural effusion. **b** Lateral projection. Cranial lobar veins (depicted by orange solid line) were considered enlarged if it was more than 0.7 times the proximal third of the 4th rib (depicted by green solid line). This cat had enlarged cranial lobar veins and arteries, severely enlarged globoid cardiac silhouette with a scant volume of pleural effusion

The assessment of mediastinum, pleural space, thoracic wall, cardiac silhouette, distribution of the pleural effusion, size of the great vessels and pulmonary vasculature, and extra-thoracic structures were adapted from previously published studies as follows. The radiologists could select “cannot assess” if they deemed it not possible to visualise a particular radiographic structure (Fig. 1). An enlarged cardiac silhouette was defined using the vertebral heart score of VHS greater than 8.1 [13, 16, 22] (Fig. 2), and/or comparisons of base to apex dimensions against the length of the sternebrae 2 to 4 [16, 23] (see Fig. 2). Cardiomegaly and left atrial dilation was also subjectively assessed based on altered cardiac shape (e.g. valentine shaped heart and slipper shaped heart [13, 16]). Pulmonary vasculature was assessed by comparing the relative size of the pulmonary artery to the pulmonary vein [8, 13, 16]. When the vessels were tortuous and did not taper at the periphery, the vasculature was assessed to be subjectively enlarged. Objectively, the pulmonary vasculature was enlarged if the cranial lobar vessels were more than 0.7 times the proximal third of the fourth rib [13, 16, 24] (Fig. 3a). The caudal lobar vessels do not have reported normals in the feline patient [16, 24] but were considered enlarged if they were more than 1:1 ratio of the 9th or 10th rib [8] at their intersection (Fig. 3b).

**Fig. 4:**
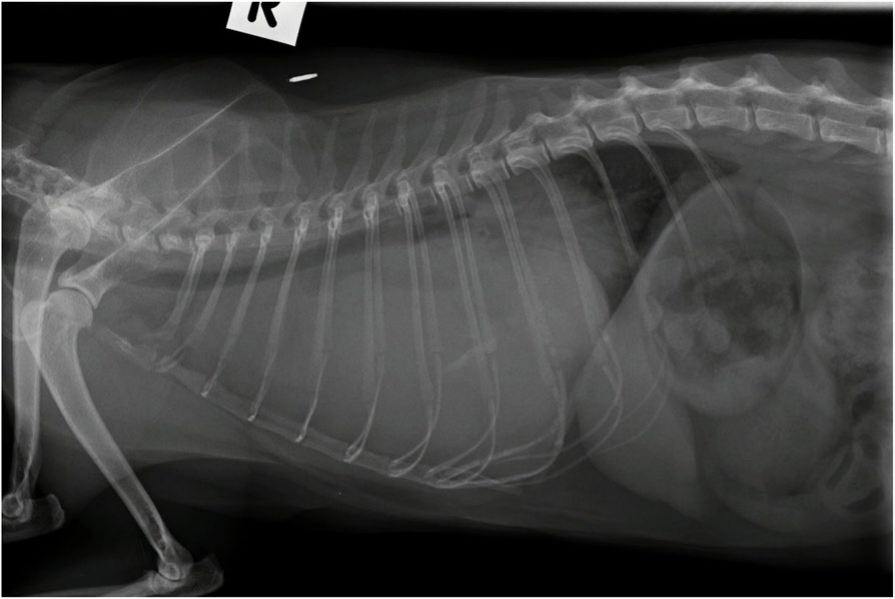
The mediastinum has a large soft tissue mass with rounded caudal and lateral margins causing severe deviation of the mediastinal structures (trachea, oesophagus) and lung lobes with complete effacement of the cardiac silhouette. This patient had mediastinal lymphoma

A mediastinal mass was suspected if there was widening of the mediastinum by a large soft tissue opacity with convex margins with deviation of mediastinal structures (trachea, oesophagus), and focal lateral and caudal displacement of the cranial lung margins from the thoracic inlet (Fig. 4). The mediastinum was unable to be assessed if there was a large volume of effusion which effaced the mediastinum and cranial lung lobes. Pulmonary parenchyma was assessed for evidence of bronchial, alveolar, unstructured or structured interstitial patterns [8, 13, 16]. Lung ‘nodules’ were defined by the authors as rounded soft tissue opacity(ies) less than one intercostal space in diameter; a lung ‘mass’ was defined as soft tissue opacity(ies) more than one intercostal space diameter. The visceral pleura was considered abnormal if there were abnormally rounded or scalloped margins, focal indentations or irregularities.

Distribution of effusions were assessed subjectively and categorised into unilateral, bilateral, asymmetrical and ‘cannot assess’. Information regarding whether radiographs were performed pre or post thoracocentesis was not available. Radiographs where a thoracostomy tube or presence of pneumothorax were not assessed for fluid distribution and volume. Left and right hemithorax effusions were recorded as ‘radiographically not detectable’, mild, moderate or severe. Mild effusions were defined as radiographs showing costophrenic angle rounding with mild scalloping, no retraction of lungs from the thoracic wall, mild scalloping on lateroventral mediastinum. Moderate effusions caused widening of interlobular fissures and slight retraction from the thoracic wall. Severe effusions caused complete effacement of the cardiac silhouette, diaphragm, and displacement of the trachea [4].

### Statistical evaluation

Positive predictive values, negative predictive values, sensitivities and specificities of radiographic signs were calculated using SPSS (SPSS Inc., NY: IBM Corp).

## Results

A total of 148 cats met the inclusion criteria. The median age at initial consultation was 9.7 years (from 0.3 – 21 years old). 85 cats (57.4%) were neutered males, 4 (2.7%) were non-neutered males, 57 (38.5%) were spayed females, 2 (1.4%) were non-neutered females. 146 patients had breed recorded; 69.5% were cross breeds and 29.5% were pedigree breeds, with 24 breeds represented. The most common breeds in the study were domestic short hair (47.3%), domestic medium hair (11.5%), domestic long hair (6.1%), and Ragdoll (6.1%).

### Prevalence of definitive diagnoses

The most common cause of pleural effusion was CHF (53.4%), followed by neoplasia (20.3%), pyothorax (10.8%) and idiopathic chylous effusion (5.4%) (Table 1). The ‘FIP’ (1.4%), ‘other’ category (4.8%) and ‘concurrent disease’ (4.1%) made up a small proportion of cases. The diagnosis of FIP cases were presumptive, as the owners of these cats did not pursue immunofluorescent staining or tissue biopsy. Both cats had fluid analysis performed which revealed a yellow, high protein exudate with a positive Rivalta test. Cats with FIP were recorded in prevalence but not used for radiographic assessment.

**Table 1:** Prevalence of disease aetiologies in 148 cats with pleural effusion

	Conditions	Number of cats	Percentage (95% CI)
	CHF	79	53.4 (49.9.0–56.9)
	Neoplasia	30	20.3 (16.8–23.8)
	Pyothorax	16	10.8 (7.3–14.3)
	Idiopathic chylothorax	8	5.4 (1.9–8.9)
	Feline infectious peritonitis	2	1.4 (0–4.8)
Multiple diagnoses	CHF and neoplasia	4	2.7 (0–6.1)
	CHF and fluid overload	1	0.7 (0–4.2)
	Neoplasia and pyothorax	1	0.7 (0–4.2)
Other diseases	Inflammatory lung disease	4	2.7 (0–6.1)
	Post-operative fluid overload	1	0.7 (0–4.2)
	Granulomatous pericarditis	1	0.7 (0–4.2)
	Ketoacidotic diabetes mellitus	1	0.7 (0–4.2)

### Radiographic interpretation

A total of 77/ 148 cats had radiographs available, however only 61 radiographic studies were of sufficient diagnostic quality with at least two orthogonal projections (Table 2). Cats with ‘other’ diagnoses were included in calculations for sensitivity, specificity, positive predictive and negative predictive values.

**Table 2:** Distribution disease for cats with radiographic studies available

	CHF	Neoplasia	Pyothorax	Idiopathic Chylothorax	Other
Number of cats	27	16	12	4	2

Radiographic findings are summarised in Table 3, sensitivity, specificity, positive and negative predictive values are presented in Table 4.

**Table 3:** Radiographic findings in cats separated into different aetiologies

Radiographic signs		CHF (*n* = 27)	Chylothorax (*n* = 4)	Pyothorax (*n* = 12)	Neoplasia (*n* = 16)	Other (*n =* 2)
Cardiac size	Enlarged	18	1	0	1	0
	Normal	3	2	5	4	2
	Cannot assess	6	1	7	11	0
Mediastinum	Normal	17	4	9	4	2
	Visible mass	0	0	0	3	0
	Cannot assess	10	0	3	9	0
Pulmonary artery cranial lobar	Normal	15	3	6	1	2
	Enlarged	2	0	0	0	0
	Cannot assess	10	1	6	15	0
Pulmonary artery caudal lobar	Normal	8	3	5	7	2
	Enlarged	13	0	5	1	0
	Cannot assess	6	1	2	8	0
Pulmonary vein cranial lobar	Normal	12	3	6	2	2
	Enlarged	4	0	0	0	0
	Cannot assess	11	1	6	14	0
Pulmonary vein caudal lobar	Normal	9	3	5	7	2
	Enlarged	11	0	4	1	0
	Cannot assess	7	1	3	8	0
Pulmonary parenchyma	Interstitial pattern	19	3	9	8	1
	Alveolar pattern	14	3	11	6	0
	Bronchial pattern	4	0	4	3	1
	Nodules	0	1	1	2	0
	Mass	0	0	0	4	0
	Cannot assess	1	0	0	1	0
Pleural margins	Normal	23	1	8	14	2
	Rounded / irregular	4	3	4	2	0

**Table 4:** Sensitivity, specificity, positive predictive values and negative predictive values of various radiographic signs in identification of different pleural effusion aetiologies in cats

Radiographic sign(s)	Final diagnosis	PPV (95% CI)	NPV (95% CI)	Sensitivity (95% CI)	Specificity (95% CI)
Enlarged heart	Neoplasia	0.05 (0.09, 0.24)	0.75 (0.62, 0.85)	0.20 (0.05, 0.72)	0.39 (0.22, 0.58)
	CHF	0.90 (0.71, 0.97)	0.81 (0.60, 0.93)	0.86 (0.64, 0.97)	0.87 (0.60, 0.98)
	Pyothorax	0.00	0.69 (0.58, 0.78)	0 (0.00, 0.52)	0.35 (0.19, 0.55)
	Chylothorax	0.05 (0.01, 0.21)	0.88 (0.74, 0.94)	0.33 (0.08, 0.91)	0.42 (0.25, 0.61)
Mediastinal mass	Neoplasia	1.00	0.79 (0.73, 0.83)	0.25 (0.07, 0.52)	1.00 (0.92, 1.00)
	CHF	0.00	0.57 (0.54, 0.60)	0.00 (0.00, 0.13)	0.89 (0.75, 0.97)
	Pyothorax	0.00	0.79 (0.78, 0.80)	0.00 (0.00, 0.26)	0.92 (0.80, 0.98)
	Chylothorax	0.00	0.93 (0.92, 0.93)	0.00 (0.00, 0.60)	0.93 (0.83, 0.98)
Pleural margins rounded	Neoplasia	0.15 (0.04, 0.42)	0.71 (0.65, 0.75)	0.12 (0.01, 0.36)	0.76 (0.60, 0.87)
	CHF	0.31 (0.13, 0.56)	0.52 (0.46, 0.58)	0.15 (0.04, 0.34)	0.74 (0.56, 0.87)
	Pyothorax	0.31 (0.14, 0.55)	0.83 (0.77, 0.88)	0.33 (0.10, 0.65)	0.82 (0.68, 0.91)
	Chylothorax	0.23 (0.12, 0.40)	0.98 (0.90, 1.00)	0.75 (0.19, 0.99)	0.82 (0.70, 0.91)
Pulmonary mass	Neoplasia	1.00	0.80 (0.75, 0.84)	0.25 (0.07, 0.52)	1.00 (0.92, 1.00)
	CHF	0.00	0.53 (0.50, 0.56)	0.00 (0.00, 0.13)	0.88 (0.72, 0.97)
	Pyothorax	0.00	0.91 (0.91, 0.92)	0.00 (0.00, 0.60)	0.91 (0.80, 0.98)
	Chylothorax	0.00	0.81 (0.80, 0.82)	0.00 (0.00, 0.26)	0.93 (0.82, 0.98)
Pulmonary nodules	Neoplasia	0.50 (0.13, 0.87)	0.76 (0.72, 0.80)	0.13 (0.02, 0.38)	0.95 (0.85, 0.99)
	CHF	0.00	0.53 (0.50, 0.56)	0.00 (0.00, 0.13)	0.88 (0.72, 0.97)
	Pyothorax	0.25 (0.04, 0.75)	0.80 (0.77, 0.83)	0.08 (0.21, 0.38)	0.94 (0.82, 0.99)
	Chylothorax	0.25 (0.04, 0.72)	0.95 (0.91, 0.97)	0.25 (0.63, 0.81)	0.95 (0.85, 0.99)
Caudal pulmonary artery	Neoplasia	0.05 (0.09, 0.26)	0.72 (0.63, 0.80)	0.13 (0.03, 0.53)	0.50 (0.33, 0.67)
	CHF	0.68 (0.5, 0.82)	0.68 (0.54, 0.79)	0.62 (0.38, 0.82)	0.74 (0.52, 0.90)
	Pyothorax	0.26 (0.15, 0.43)	0.80 (0.67, 0.89)	0.50 (0.19, 0.81)	0.59 (0.41, 0.75)
	Chylothorax	0.00	0.88 (0.85, 0.91)	0.00 (0.00, 0.71)	0.54 (0.37, 0.69)
Caudal pulmonary vein	Neoplasia	0.06 (0.01, 0.30)	0.73 (0.65, 0.80)	0.13 (0.00, 0.53)	0.56 (0.38, 0.73)
	CHF	0.69 (0.48, 0.84)	0.65 (0.53, 0.76)	0.55 (0.32, 0.77)	0.77 (0.55, 0.92)
	Pyothorax	0.25 (0.12, 0.44)	0.81 (0.43, 0.74)	0.44 (0.14, 0.79)	0.64 (0.45, 0.80)
	Chylothorax	0.00	0.88 (0.86, 0.91)	0.00 (0.00, 0.71)	0.59 (0.42, 0.74)
Alveolar pattern	Neoplasia	0.18 (0.10, 0.29)	0.64 (0.50, 0.76)	0.40 (0.16, 0.68)	0.36 (0.23, 0.52)
	CHF	0.41 (0.31, 0.52)	0.48 (0.33, 0.63)	0.54 (0.33, 0.73)	0.35 (0.19, 0.55)
	Pyothorax	0.32 (0.25, 0.40)	0.96 (0.78, 0.99)	0.92 (0.62, 1.00)	0.51 (0.36, 0.66)
	Chylothorax	0.09 (0.5, 0.15)	0.96 (0.81, 0.99)	0.75 (0.19, 0.99)	0.44 (0.30, 0.58)
Bronchial pattern	Neoplasia	0.25 (0.09, 0.52)	0.74 (0.68, 0.80)	0.20 (0.04, 0.48)	0.80 (0.65, 0.90)
	CHF	0.33 (0.14, 0.60)	0.53 (0.47, 0.59)	0.15 (0.04, 0.35)	0.76 (0.58, 0.89)
	Pyothorax	0.33 (0.15, 0.58)	0.83 (0.76, 0.88)	0.33 (0.10, 0.65)	0.83 (0.69, 0.92)
	Chylothorax	0.00	0.91 (0.90, 0.93)	0.00 (0.00, 0.60)	0.78 (0.65, 0.88)
Interstitial pattern	Neoplasia	0.20 (0.13, 0.29)	0.63 (0.45, 0.78)	0.53 (0.27, 0.79)	0.27 (0.15, 0.43)
	CHF	0.48 (0.39, 0.56)	0.63 (0.44, 0.79)	0.73 (0.52, 0.88)	0.36 (20.4, 0.55)
	Pyothorax	0.23 (0.16, 0.30)	0.84 (0.65, 0.94)	0.75 (0.43, 0.95)	0.34 (0.21, 0.49)
	Chylothorax	0.08 (0.04, 0.13)	0.95 (0.76, 0.99)	0.75 (0.19, 0.99)	0.33 (0.21, 0.47)

*PPV* positive predictive values, *NPV* negative predictive values, *CHF* congestive heart failure, *PV* pulmonary vein, *PA* pulmonary artery

An enlarged cardiac silhouette (20/61) was most often identified in cats with congestive heart failure (18/20). Cardiomegaly was also identified in one cat with neoplasia (1/20) and one cat with chylothorax (1/20). The positive predictive value of cardiomegaly for congestive heart failure was 90%, and had a negative predictive value of 81%, sensitivity of 86% and specificity of 87%. The positive predictive value of cardiomegaly for neoplasia and chylothorax was 5% and pyothorax was 0%.

A radiographic mediastinal mass (3/61) was only identified in cats with neoplasia. In the neoplasia group 3/16 (18.8%) of cats had a radiographic mass. 4/16 (25%) cats with neoplasia had a radiographically normal mediastinum and 9/16 (56.3%) cats were interpreted to have fluid effacing the mediastinum leading to an inability to assess this structure. For the prediction of neoplasia, presence of a mediastinal mass had a positive predictive value of 100%, negative predictive value of 79%, sensitivity of 25% and specificity of 100%.

Pulmonary mass-like lesions (4/61) were only identified in cats with neoplasia. Pulmonary masses had a 100% positive predictive value for neoplastic disease, negative predictive value of 80%, sensitivity of 25% and specificity of 100%. Pulmonary nodules were identified in a small number of cats (4/61), including two cats with neoplasia, one cat with pyothorax and one cat with chylothorax. No cats with congestive heart failure had radiographic masses or nodules. The positive predictive value was 50% for neoplastic disease, 25% for pyothorax and 25% for chylothorax. Sensitivity was low (0%) but specificity was high for pyothorax (91%) and chylothorax (93%).

Cranial lobar arteries and veins were often difficult to assess due to marked fluid effacement therefore, only caudal lobar arteries and veins were utilised in calculations. Pulmonary artery and venous enlargement had a 68% and 69% positive predictive value for congestive heart failure with a sensitivity of 62% and 55% respectively and a specificity of 74% and 77%. Vascular enlargement had a low positive predictive value, high negative predictive value, and low sensitivity and specificity for pyothorax, neoplasia and chylothorax.

Almost all cats (59/61) cats had radiographically abnormal pulmonary parenchyma. The remaining two cats had marked pleural effusion and the parenchyma could not be assessed. Interstitial and alveolar pattern were most frequently identified. The pulmonary patterns were not predictive for any specific disease with under 50% positive predictive value for all aetiologies. Similarly, few cats had abnormal or rounded pleural margins and this radiographic sign alone was poorly predictive (under 31%) for any aetiology.

Fifteen cats either had a thoracocentesis drain in situ or had evidence of pneumothorax consistent with recent thoracocentesis (Table 5). Where laterality of effusion was detectable, all cats without a thoracic drain and pneumothorax had bilateral effusion (42/42). 10/42 cats had bilateral but asymmetrical distribution of effusion between the left and right hemithoraces.

**Table 5:** Radiographic distribution and volume of fluid in cats without pneumothorax and thoracostomy tube

Radiographic signs		Cardiac (*n =* 25)	Chylothorax (*n =* 1)	Pyothorax (*n* = 3)	Neoplasia (*n* = 15)	Other (*n =* 2)
Pneumothorax		2	3	4	0	0
Thoracostomy tube present		0	0	6	1	0
Distribution	Asymmetrical	5	0	0	5	0
	Bilateral	22	1	3	15	1
	Fluid not detectable	3	0	0	0	0
	Cannot assess	1	0	0	0	1
Left hemithorax	Fluid not detectable	3	1	0	0	0
	Mild	12	0	0	6	1
	Moderate	7	0	2	3	0
	Severe	3	0	1	6	0
	Compartmentalised	0	0	0	0	0
Right hemithorax	Fluid not detectable	3	0	1	0	1
	Mild	9	1	0	5	1
	Moderate	9	0	1	5	0
	Severe	3	0	1	4	0
	Compartmentalised	0	0	0	1	0

## Discussion

The present study is the first to examine cats with pleural effusions and the positive and negative predictive values of radiographic signs for common underlying aetiologies. In our sample of cats with pleural effusion, over half were caused by congestive heart failure, and the other half comprised of neoplasia, pyothorax, idiopathic chylous effusion, by a combination of causes or due to ‘other’ uncommon causes. This is consistent with recent studies [2, 3, 25] that have identified congestive heart failure and neoplasia as the most frequent underlying cause of pleural effusion. The results are in contrast to previous retrospective studies [26–28] which identified that pleural effusions were more likely to be caused by neoplasia, pyothorax or FIP.

This study found that a radiographically enlarged cardiac silhouette and concurrent pleural effusion should prompt the suspicion of CHF with a 90% positive predictive value. The finding of cardiomegaly in cats with congestive heart failure is not unexpected, as volume overload is the basis of the pathophysiology in congestive heart failure [12, 13, 16, 22] and is consistently reported. Although a small percentage of our cats with congestive heart failure (3/27) did not have radiographic cardiomegaly, the negative predictive value (81%) and specificity (87%) remained high. These three cases were diagnosed with hypertrophic cardiomyopathy, which can be radiographically silent [13]. Dehydration and frusemide administration could be a reason for the normal cardiac size [13, 22]. One cat had radiographs performed post-frusemide therapy and another had concurrent renal failure with concern for clinical dehydration. No information on treatment was available for the third patient. Our results reflect similar results to a previous study [13] that found a small number (4/100 cats) with heart failure did not show enlarged cardiac silhouette on radiography with confirmation on echocardiography. In our study, two patients with radiographic cardiomegaly were diagnosed with neoplasia and chylothorax. The cause of cardiomegaly in these cases could be due to volume or pressure overload from iatrogenic or intravenous fluid overload [8] or reduced cardiac output from bradycardia, or severe anaemia [29]. Anaemia may lead to reduced tissue oxygenation causing complex haemodynamic compensatory mechanisms leading to peripheral vasodilation and promotion of sodium and water retention and volume overload [29]. Unfortunately, information on haematocrit or packed cell volume was not available for these two cases and the cause of cardiomegaly is speculative.

Echocardiography remains the gold standard for definitive diagnosis of congestive heart failure; however, if echocardiography is not available, other tests such as NT-proBNP are recommended as an initial diagnostic test when pleural effusion is identified [30]. Cardiac biomarkers such as NT-proBNP can assist in differentiating cardiac versus non-cardiac disease through measurement of plasma [31] or pleural effusion levels [32]. NT-proBNP is useful when comprehensive echocardiography is not available, or when there may be concurrent disease (i.e., cardiac disease and neoplasia) or for monitoring of cardiac disease progression [30].

Radiographic signs of congestive heart failure in cats can be highly variable, which may include the absence or presence of interstitial or alveolar pulmonary pattern, bronchial pattern, enlargement of pulmonary vessels and pleural effusion [16, 22, 33, 34]. Pulmonary arterial and venous enlargement only had a moderate (68% to 69%) positive predictive value for cats with CHF in the current study. This is supportive of previous studies, which found only half to two thirds of cats in congestive heart failure had venous or arterial enlargement [13]. In our study, venous and arterial enlargement were observed across all causes of pleural effusion, and vascular size was not sensitive or specific for any particular aetiology. Pulmonary arterial and venous dilation can occur secondary to a variety of causes and are often dynamic with rapid change in diameter depending on intraluminal pressure and volume [35]. Dehydration from diuretics can lead to reduced vessel size [13, 22] and increased vessel size can be caused by volume or pressure overload [13, 22], thromboembolic diseases [35] or chronic lung diseases [8]. There are few objective measurements of normal pulmonary vessel size in the literature [16, 24] and defining normal and abnormal caudal pulmonary arterial and venous size in cats are often performed under the subjective opinion of the observers [12, 13]. To the author’s knowledge, there is only one report describing abnormal caudal pulmonary artery in cats with heart worm disease [36], with pulmonary arteries considered abnormal if they were larger than 1.6 times the width of rib 9th at its intersection. In our study, the radiologists encountered issues with interpreting the size of the vessels based on the lack of objective data and found the caudal pulmonary artery and vein more clearly intersected rib 10 rather than rib 9 in many cases. Vascular enlargement in this study was defined as the vessels exceeding more than one times the dimension of the 10th rib at its intersection.

In our sample of cats, interstitial, alveolar and bronchial patterns were non-specific at predicting any aetiology. This is not a surprising finding as it is well known that lung tissue can only respond to injury in a limited number of ways leading to overlap in radiographic changes and pulmonary patterns across a wide range of disease (exudate, cellular, oedema, haemorrhage) [4, 8, 37]. In cats, cardiogenic oedema distribution is well reported to have a highly variable appearance [16, 22, 33].

In the literature, there are varying reports [1–4, 7] on whether neoplastic, chylous and pyothorax effusions cause unilateral or bilateral effusions. It is postulated that the mediastinum is fenestrated in cats [4]; however, excessive fibrin may seal the mediastinum [8] (i.e. caused by rupture of abscess, unilateral pleuritis causing adhesions etc). Previous reports [4, 6] describe chylothorax and pyothorax to have unilateral effusions due to the highly inflammatory nature of the effusion. Studies by Barrs and Beatty [38] and others [9, 28, 39, 40], identified that most cats with pyothorax (70–90%) had bilateral effusions. Our results found that all cats with a radiographically identifiable pleural effusion had bilateral effusions regardless of disease aetiology. The limitation to this finding is that our sample had a low number of cats with chylothorax (1/46) and pyothorax (3/46) which may lead to under-representation of radiographic presentations in cats with these two conditions.

The presence of a radiographic mediastinal mass had 100% positive predictive value for neoplasia with a low sensitivity and high specificity. When a mediastinal mass was radiographically diagnosed, there was a high probability of neoplastic disease; however, not all neoplastic diseases in our study were caused by mediastinal abnormalities, which is reflected in the low sensitivity. A total of 7/12 cats with neoplasia had cytologically or histologically confirmed cranial mediastinal masses, but only 3/7 cats were assessed to have a radiographic mediastinal mass. The mediastinum could not be assessed by the radiologists in the remainder 4/7 cats thus leading to over half the cats with mediastinal masses not radiographically identified. In retrospect, the radiologists both agreed that on occasions, they had interpreted the mediastinal mass as loculated pleural effusion. The presence of effusion and mediastinal masses both obscured the region of the mediastinum, and caused displacement of the cranial lung lobes and elevation of the trachea [8]. These overalapping radiographic signs lead to difficulty interpreting the changes in the mediastinum, thus leading to a high number of mediastinal masses not identified on radiographs. It should however be noted that radiographic mediastinal masses were not identified in any other aetiology; therefore, if a radiographic mediastinal mass is suspected, it may be worth using a different diagnostic modality to rule out a neoplastic process affecting the mediastinum (i.e. ultrasound, CT, sampling).

Radiographic pulmonary nodules only predicted neoplastic disease 50% of the time. Pulmonary nodules were observed in one cat with pyothorax and chylothorax. Various non-neoplastic diseases may lead to pulmonary soft tissue nodules, including bronchial disease [41], granulomatous diseases (fungal, bacterial, abscesses) [8, 42], toxoplasma [43], *Aelurostrongylus abstrusus* [44] or enlarged end-on vessels [8]. No CT or postmortem was performed and the cause of nodules in the cat with pyothorax and chylothorax is not definitively known but could be associated with concurrent inflammatory or infectious diseases or could be incidental and artefactual or from focal regions of atelectasis.

**Fig. 5:**
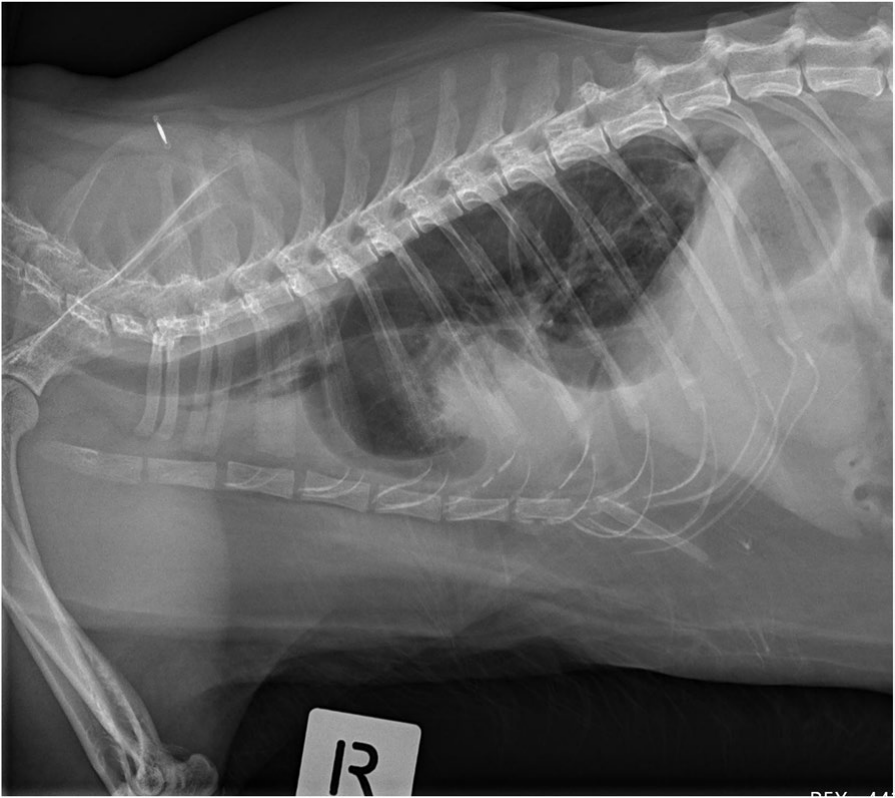
Rounded and contracted pleural margins secondary to chronic fibrosis is often described in cats with pyothorax. This cat represents the classic chronic chylothorax pleural margin abnormalities; however, this was not a predictive sign of chylothorax in our cohort

In previous studies, cats with chylothorax have been described to have rounded pleural contours due to chronic fibrosing reaction of visceral pleura [9, 11] (Fig. 5), exerting a constrictive and restrictive effect on the lung lobes. It was found that pleural abnormalities were not useful as a predictor, and only had a 23% positive predictive value for chylous effusion. This suggests that chylothorax may not always cause a constrictive and fibrosing effect to the pleura; however, the sample size examined was small and these results may not generalise to other populations.

There are many limitations to this study, particularly pertaining to the retrospective access to cases. Only 61/148 cases had radiographs that were available for assessment which could have created bias from missing data from the excluded cats. An effort was made to select cases that had diagnostic radiographs and as not all cats had thoracic radiographs as part of the diagnostic work up to achieve the final diagnosis. These exclusions may lead to significant over-representation or under-representation of specific diseases and specific radiographic signs. In addition, the study was performed using only two radiologists that read the studies via consensus. Although many radiographic signs were pre-defined and read with objectivity, there remained a degree of subjective interpretation over several radiographic signs (i.e. presence or absence of a mediastinal mass). This may lead to differences in opinion or radiographic conclusions when interpreted by other readers. Finally, despite collecting data across 10 years, the sample size is small, particularly for the non-CHF groups, which may cause the lack of precision in the predictive values, sensitivity and specificity estimates and lead to lowered statistical power in our findings. In particular, feline infectious peritonitis was not well represented in our cohort. Although this could reflect true prevalence, it may also reflect under-representation. The data collected was prior to the availability of broad spectrum coronavirus protease inhibitor GC376 [45], when FIP was considered fatal and many owners were declining further diagnostics once a suspicion was established. A larger scale study examining the radiographic features in more cases would allow for more accurate results, therefore although this study is novel for examining radiographic features of cats with pleural effusions, it is preliminary.

We have accounted for multiple diseases in a single patient, it is possible that some cats had more than one disease causing pleural effusion that we were unaware of, with a potential for misclassification bias which may also affect the appearance of radiographs. For example, cats with congestive heart failure but also neoplastic disease may have radiographic features of both diseases.

All cases were obtained in practices in metropolitan Western Australia and the practices were within a 10 km radius from the referral hospital. When interpreting the data, clinicians should be aware of the effects of disease prevalence on test results, which may affect the accuracy of the results, as the PPV and NPV results assume the prevalence of the disease aetiologies are similar in all populations.

## Conclusions

In our sample of cats with pleural effusions, cardiomegaly is the most reliable predictor of CHF; mediastinal masses may predict neoplastic disease. Radiography alone is helpful but inadequate at differentiating underlying disease aetiologies, particularly for idiopathic chylothorax and non-mediastinal neoplasms as these diseases have non-specific radiographic signs.

## Supplementary Information

**Additional file 1.**

## Data Availability

All data generated or analysed during this study are included in this published article (and its supplementary information files).

## Abbreviations

CHF: Congestive heart failure
CT: Computed tomography
FIP: Feline infectious peritonitis
PPV: Positive predictive values
NPV: Negative predictive values
PV: Pulmonary vein
PA: Pulmonary artery
